# Surgery of the Stomach

**Published:** 1906-01-13

**Authors:** 


### SURGERY OF THE STOMACH.

**Gastro-enterostomy and Pyloroplasty** are the two most prominent methods for the relief of non-malignant pyloric obstruction. The principles underlying these operations are much better understood than they were a few years ago, but Cannon and Mahe[1] have carried out an important experimental study by means of the Röntgen rays which throw some important light on the subject. According to them the stomach must not be regarded as a passive bag. During digestion the cardiac end slowly contracts, pressing its contents into the pyloric end. Observations on the functioning human stomach show that as it empties it shortens, especially along the greater curvature. Therefore the part of the stomach lowest when the organ is full or relaxed is not lowest as it empties. The pylorus then becomes the lowest point. Even if "gravity drainage" occurred, the pylorus is the natural outlet so long as the stomach retains its power of contracting. The pressure within the abdomen is approximately atmospheric pressure. The pressure in any part of the passive alimentary canal depends on the weight of the overlying abdominal organs. If the canal is inactive, the food therefore is as if surrounded by water. Gravity cannot act, and gravity drainage does not occur. Because peristaltic waves move towards the pylorus, the intragastric pressure is three or four times greater at the pylorus than in the cardiac end. Observation on large cats with gastro-enterostomy openings of various sizes and at various parts of the stomach showed that unless the opening, or stoma, was in the antrum (*i.e.*, close to the pylorus) the food, even when fluid, was pushed through the pylorus rather than through the stoma. Circulation of food through the pylorus to the duodenum and back to the stomach through the anastomosis was repeatedly observed, but it was not followed by the clinical symptoms of "vicious circle." The clinical symptoms of the "vicious circle" were observed in animals in which a kink of the intestine was found just distal to the anastomosis. Kinks at this point cannot be straightened by peristaltic activity because the circular fibres of the intestine are cut at the neighbouring stoma. It is important that food be mixed with the secretions poured into the duodenum: these juices are highly effective in digestion, and also neutralise the acid chyme. If food leaves the stomach by the stoma, it is not mixed with these secretions. Jejunal ulcers occurring after gastro-enterostomy may be due to the presence of acid in a region in which inorganic acid is not normally found. From the above considerations it was concluded that the stoma should be large and as near to the pylorus as possible; that the circulation of the food be rendered less probable by avoidance of over-eating; and that so far as possible kinks be obviated by attaching a narrow band of the distal gut to the stomach for several centimetres beyond the stoma, thus permitting peristalsis to become an effective force. The probability of a circulation of the food whenever the pylorus is left open, the non-mixture of the food with the digestive and neutralising fluids in the duodenum, and the ever-present danger from kinks in gastro-enterostomy make the operation not an ideal one. In pyloroplasty (preferably the Finney operation) these objections are avoided, and a too rapid exit of the food through the pylorus is prevented by rhythmic segmentation of the food in the duodenum —an activity which in part replaces the functions of the pylorus, and also mixes the food with the pancreatic juice and the bile. Sinclair White[2] and others consider that gastro-duodenostomy is a desirable substitute for gastro-jejunostomy in cases of pyloric obstruction, and it is claimed that the former operation is not subject to the defects which accompany the latter operation. Gastro-duodenostomy can be performed either by Kocher's or Finney's method. Kocher pointed out that the superior flexure of the duodenum, owing to its attachment to the right margin of the gastro-hepatic omentum containing the portal vein, hepatic artery, and common bile-duct, is incapable of being freed to any great extent, but that the whole of the descending portion, together with the inferior flexure, can be made freely movable. To effect the freeing of these portions of the duodenum he divides the peritoneum over the front of the right kidney one inch away from and parallel to the right margin of the duodenum. The fingers are inserted behind the duodenum, and it, together with the head of the pancreas, are drawn forwards and to the left. An anastomosis is then established between the descending duodenum and the right side of the curvature of the stomach. This may be called lateral gastro-duodenostomy, and Kocher announces his intention of using it in all cases of pyloric obstruction except where dense adhesions exist. It does not, however, provide so dependent an opening for drainage as is given by gastro-jejunostomy in a much dilated stomach. Finney's method, or sub-pyloric gastro-duodenostomy, is commenced by dividing adhesions around the pylorus, and freeing as much as possible the pyloric end of the stomach and the upper portion of the duodenum. A suture, to be used as a retractor, is passed through the musculo-peritoneal walls of the pylorus on its upper surface. A second similar suture is inserted into the anterior wall of the stomach, and a third into the anterior wall of the descending duodenum at equidistant points—twelve centimetres from the pyloric suture. Traction on the pyloric suture upwards and on the other two downwards facilitates the subsequent steps of the operation. The contiguous surfaces of the stomach and duodenum are then sutured together by a silk suture. It begins at the lower border of the pylorus, and is continued downwards for two, three, or more inches, according to the necessities of the case. After being tied its end is left long and secured by forceps. A horse-shoe-shaped incision is now made around the upper end of the silk suture, and downwards a quarter of an inch away from it on either side through all the coats of the stomach and duodenum. The ends of the incision should terminate half an inch above the lower limit of the silk suture. In cases where the lower half of the pylorus is thickened and indurated some of the tissues may be excised with advantage. A continuous cat-gut suture, engaging all the coats of the stomach and intestine, is commenced at the lower margin of the pylorus, and carried downwards, uniting their contiguous (posterior) edges. It is then carried forwards and upwards, uniting their outer (anterior) edges. The operation is completed by continuing the silk suture round the lower margin of the anastomosis and upwards to a point half an inch above the incision in the pylorus. Of the two operations Kocher's is the more generally applicable; but for cases where the pylorus is not buried in dense adhesions, nor greatly indurated, nor yet the seat of active ulceration, Finney's procedure appears to be an excellent one.

[1] Annals of Surg., May, 1905, p. 686. [2] Brit. Med. Jour., Oct. 7, 1905, p. 861.

